# Macular hypoplasia and high myopia in 48, xxyy syndrome: a unique case of 48, xxyy syndrome that presents with high myopia and macular dysplasia

**DOI:** 10.1186/s12886-024-03456-z

**Published:** 2024-04-23

**Authors:** Aohan Hou, Xinyu Liu, Limei Sun, Xiaoyan Ding

**Affiliations:** State Key Laboratory of Ophthalmology, Zhongshan Ophthalmic Center, Guangdong Provincial Key Laboratory of Ophthalmology and Visual Science, Sun Yat-sen University, 510060 Guangzhou, China

**Keywords:** 48, XXYY syndrome, High myopia, Macular dysplasia

## Abstract

**Background:**

Among sex chromosome aneuploidies, 48, XXYY syndrome is a rare variant. This condition is marked by the existence of an additional X and Y chromosome in males, leading to a diverse range of physical, neurocognitive, behavioral, and psychological manifestations. Typical characteristics include a tall stature and infertility. Other phenotypes include congenital heart defects, skeletal anomalies, tremors, obesity, as well as the potential for type 2 diabetes and/or peripheral vascular disease.

**Case presentation:**

A 6-year-old boy, who had been experiencing progressive vision deterioration in both eyes for the past two years, presented with a history of poor vision, delayed motor skills. The patient was diagnosed with micropenis in the pediatric outpatient clinic. Sparse hair, an unusually tall stature and craniofacial dysmorphology characterized by ocular hypertelorism, depressed nasal bridge, and epicanthic folds were observed. Comprehensive ophthalmic examination revealed high myopia and grade 3 macular hypoplasia. Diagnostic investigations including karyotype analysis and whole-exome sequencing identified an anomalous male karyotype comprising two X and two Y chromosomes, confirming a diagnosis of 48, XXYY syndrome.

**Conclusions:**

This study underscores the rare association of high myopia and grade 3 macular dysplasia with 48, XXYY syndrome. To our knowledge, this case marks the first recorded instance of macular dysplasia in a patient with 48, XXYY syndrome. This novel finding enhances our understanding of this syndrome’s phenotypic variability.

## Introduction

Among sex chromosome aneuploidies, 48, XXYY syndrome is a rare variant. This condition is marked by the existence of an additional X and Y chromosome in males, leading to a diverse range of physical, neurocognitive, behavioral, and psychological manifestations. Typical characteristics include a tall stature and infertility. Other phenotypes include congenital heart defects, skeletal anomalies, tremors, obesity, as well as the potential for type 2 diabetes and/or peripheral vascular disease [1]. Notably, the syndrome’s phenotypic presentation does not typically include ocular abnormalities. In this report, however, we introduce an exceptional case of 48, XXYY syndrome that presents with high myopia and macular dysplasia.

## Case report

A 6-year-old boy suffered from progressive deterioration of vision bilaterally during the previous 2 years. His past ophthalmic history included poor vision since early childhood and motor development delays (holding head up at 6–7 months old, walking at 1.5 years old). The patient was diagnosed with micropenis in the pediatric outpatient clinic. Both of his parents were emmetropic without any visual or systemic medical disorders. No other family history was noted. Whole Exome Sequencing was conducted on the patient and their parents, with chromosomal karyotype analysis being exclusively performed on the patient. No significant abnormalities were observed in the parents’ results.

**Fig. 1:**
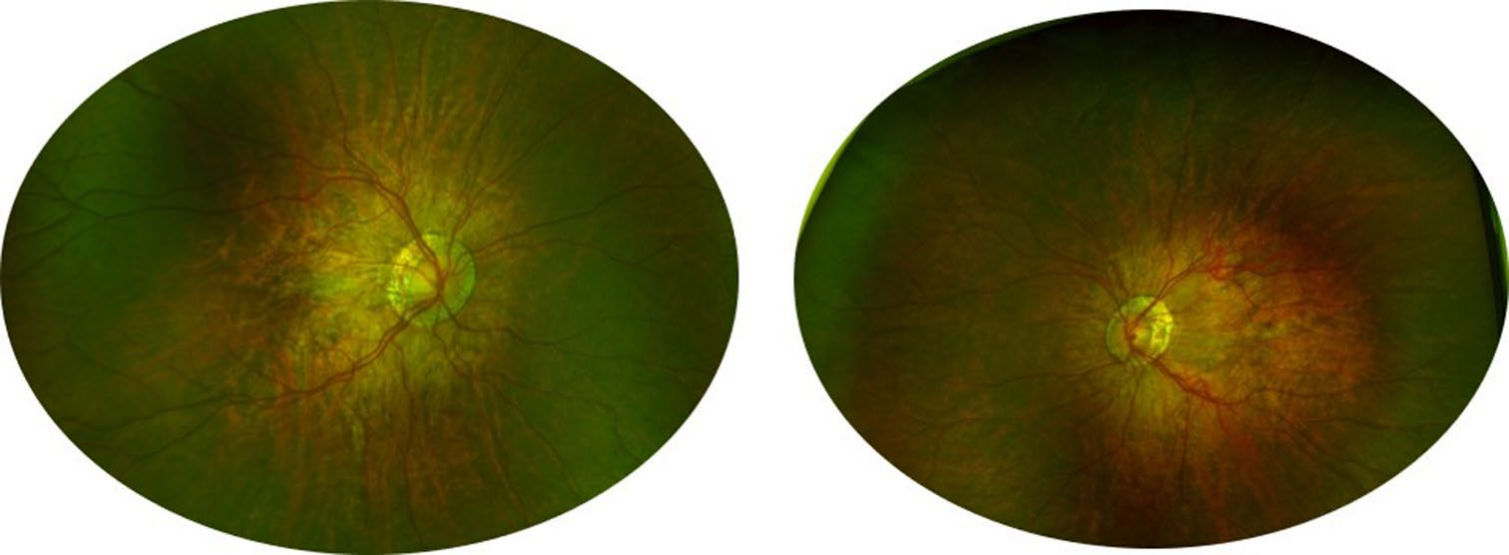
Fundoscopy showed tessellates fundus, peripapillary and posterior staphyloma.

Physical examination revealed tall stature (127 cm) compared to the normal value of 117.4 ± 5.0 for 6-year-old boys in Han [3]. The patient is present with craniofacial dysmorphology characterized by ocular hypertelorism, depressed nasal bridge, and epicanthic folds. Sparse hair was observed in the occipital region. Cycloplegic refraction revealed Bilateral high myopia and astigmatism (RE: -9.75DS/-3.50DC*177°, LE: -12.50DS/-3.50DC*12°). Best corrected visual acuity (BCVA) was LogMAR 0.4 (Snellen 20/50) in the right eye and LogMAR 0.54 (Snellen 20/70) in the left, respectively. Anterior segment was unremarkable. Vitreous Liquification and tessellated fundus were noted. The optic disc was surrounded by a ring of chorioretinal atrophy. In addition, multiple thin emanating vessels and posterior staphyloma centered with the optic disc were observed. (Fig.1).

**Fig. 2:**
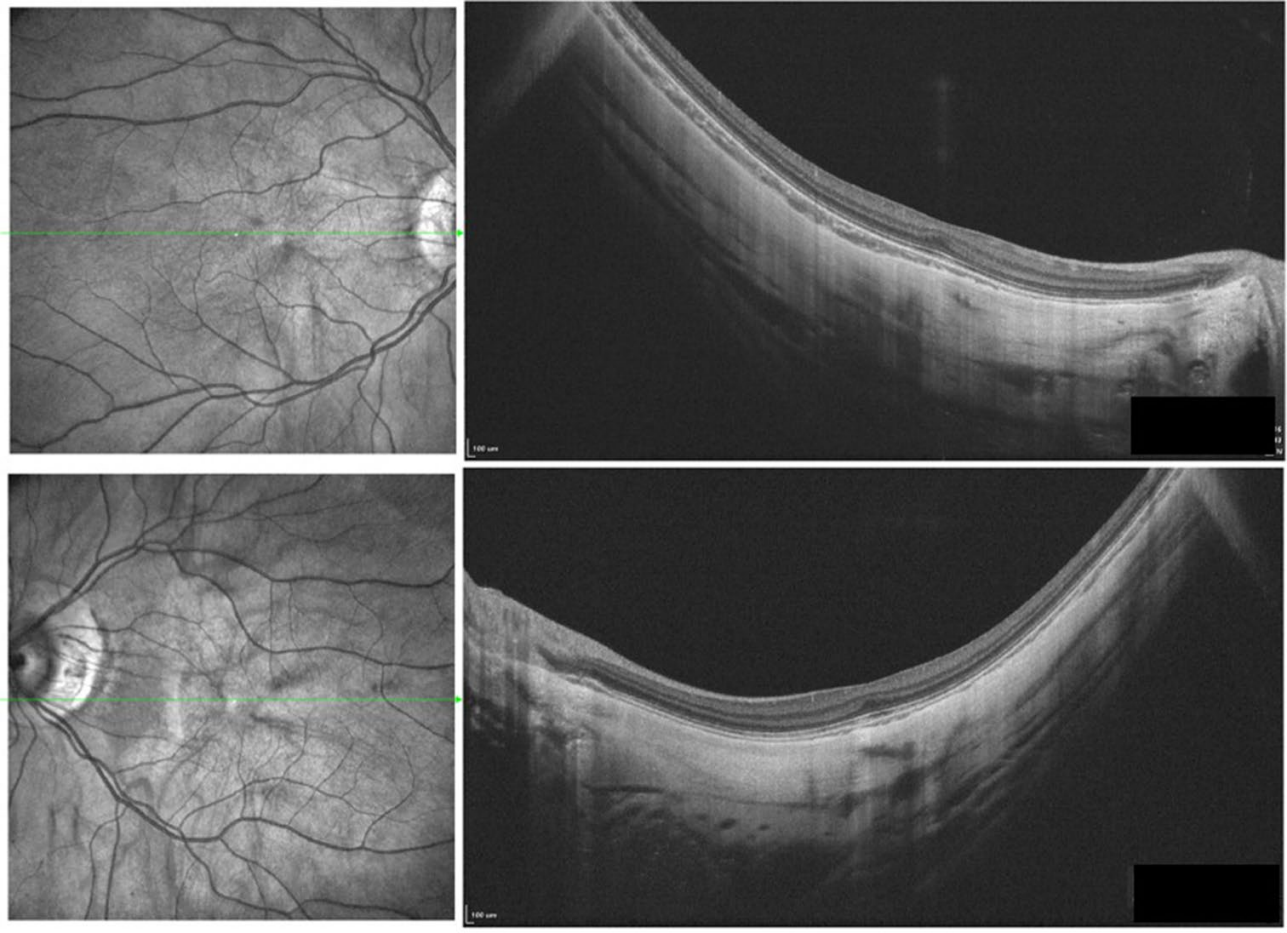
OCT showed grade 3 macular hypopiasia, extreme choroidal atrophy and staphyloma formation bilaterally.

**Fig. 3:**
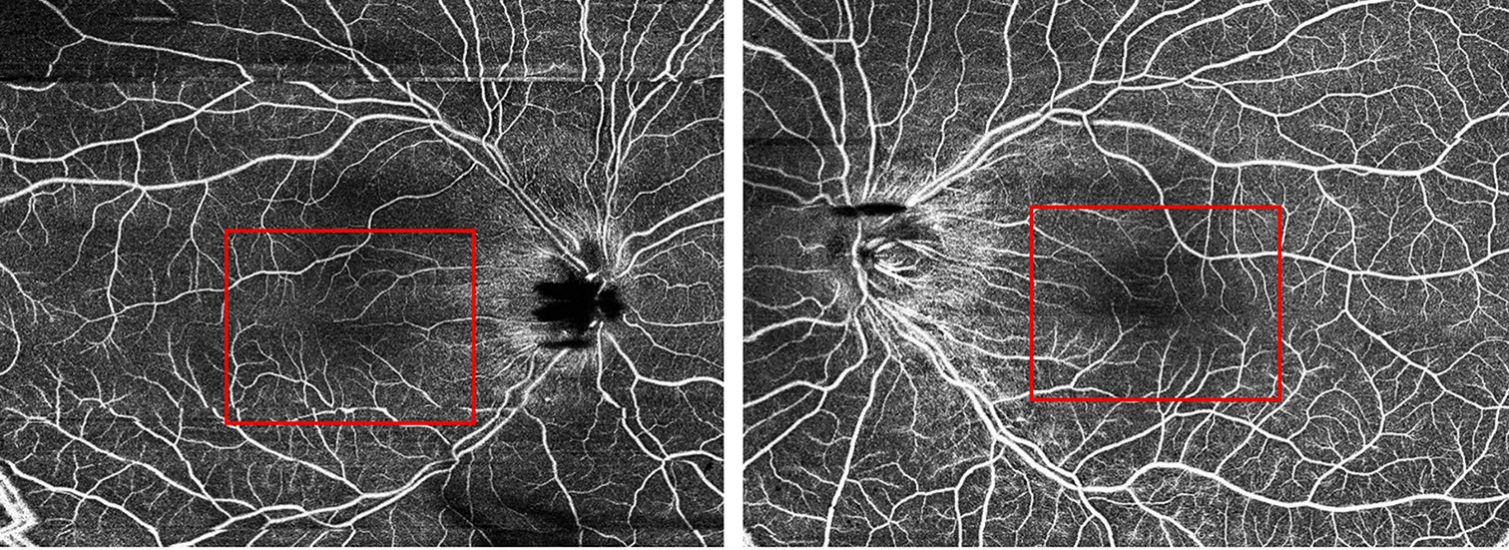
Optical coherence tomography (OCTA) showed increased vessel density with reduction of the foveal avascular zone bilaterally.

**Fig. 4:**
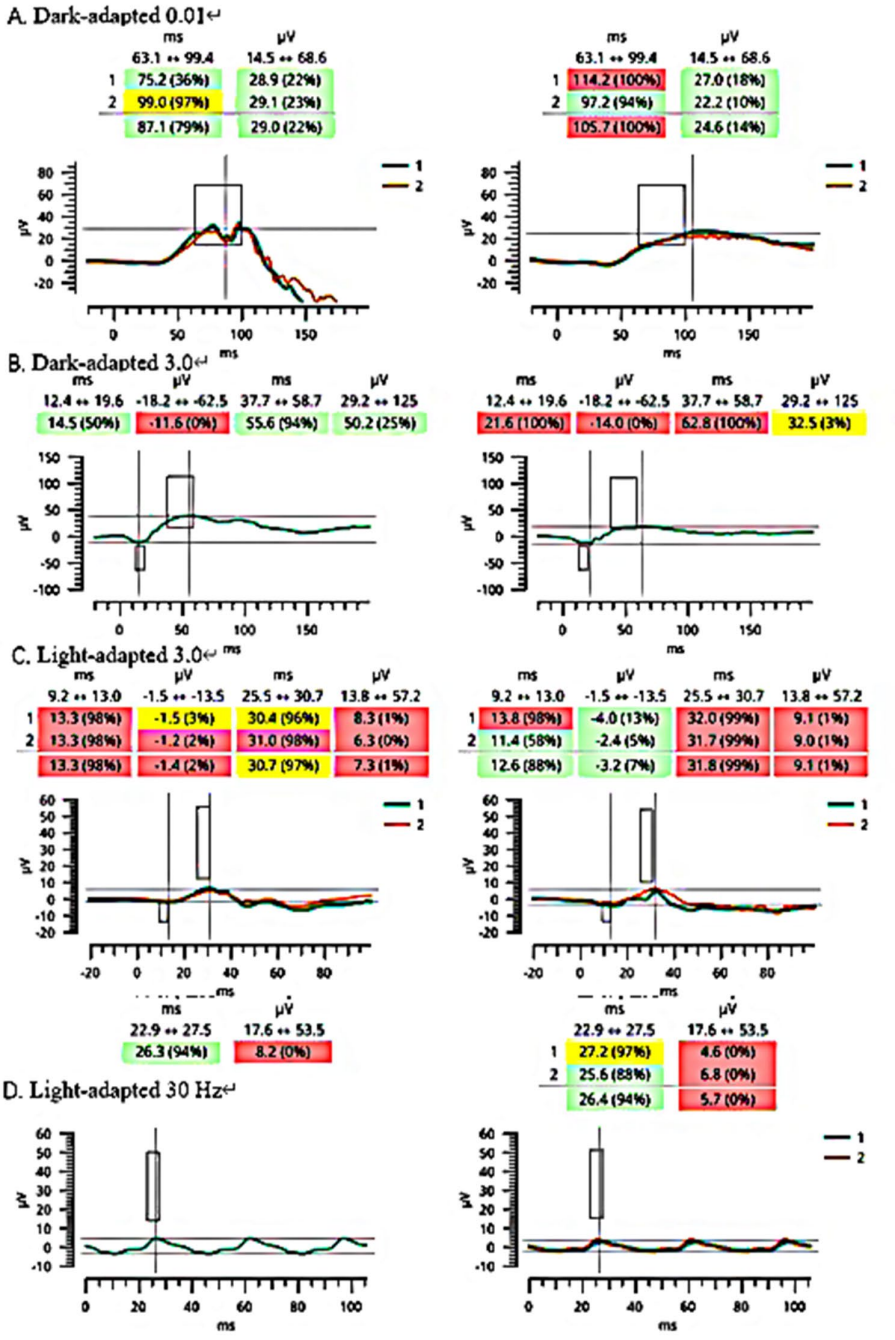
Dark-adapted 0.01 ERG and Dark-adapted 3.0 ERG ahowed allghtiy peak time delay on the right eye (**A-B**). Light-adapted 3.0 ERG and Light-adapted 30 Hz ERG showed algnificantli reduced amplltude bllaterally, while peak time delay on the right eye (**C-D**).

Grade 3 macular hypoplasia, in detail, absence of foveal pit, extrusion of the inner retina layer and lengthening of the outer segment, was noted (Fig.2) [12]. Choroidal atrophy and staphyloma were noted in both eyes. Subfoveal choroidal thickness was 40 μm in the right eye and 38 μm in the left eye. Optical coherence tomography angiography (OCTA) showed increased vessel density with reduction of the foveal avascular zone (Fig.3). Axial lengths were 28.88 mm (OD) and 29.59 mm (OS). Significant reduced amplitudes were observed on light-adapted electroretinography (ERG) and light-adapted 30 Hz ERG bilaterally (Fig.4).

Karyotype analysis were performed in accordance with the ISCN 2016, revealed an abnormal karyotype with two X chromosomes and two Y chromosomes in all cell lines consistent with 48, XXYY syndrome.

The ocular phenotype and systemic presentations were not consistent with other causes of foveal hypoplasia and high myopia, such as optic nerve hypoplasia, familial exudative vitreoretinopathy, stickler syndrome, retinopathy of prematurity, and albinism.

## Discussion

48, XXYY syndrome, as a rare sex chromosome disorder, was first described in 1964. It is estimated to occur in 1:18,000–1:40,000 male births. It presents with a wide variety of physical, psychosocial, and neurocognitive findings. Most of the literature currently focuses on the physiological and psychological disorders of the 48, XXYY phenotype. The wide spectrum of symptom variability is considered to be related to sex chromosome dosage and skewed X-inactivation, but further investigation is still needed to understand these underlying genetic variations and their connection to clinical signs and symptoms [1].

There are few reports of ocular abnormalities in association with 48, XXYY syndrome with only three known cases to date that detail ocular complications, including strabismus, Duane’s syndrome, and retinal dysfunction [5, 11, 13]. Only a single case has been reported wherein high myopia was associated with 48, XXYY syndrome [5]. In that case, bilateral poor visual acuity (BCVA LogMAR 1.0, Snellen 20/200) was found, which was align with the findings from our patient. Notably, although our patient did not exhibit the bilateral extinguished ERG response documented in the previous report [4, 7]. there was a significantly reduced amplitude in light-adapted and flicker ERG.

In this study, we present the case to describe macular dysplasia in a patient with 48, XXYY syndrome. The absence of the foveal pit, protrusion of the inner retinal layer, and elongation of the outer segment were noted on optical coherence tomography with increased vessel density with reduction of the foveal avascular zone on OCTA. Chen etc. reported a case of macular dysplasia with 48, XXYY syndrome. In their study, the degree of macular hypoplasia in patients was less severe than in our case, with better visual acuity, and there was no description of the refractive status [2]. The severity of macular dysfunction and poor BCVA was correlated with the degree of macular dysplasia and high myopia observed. Posterior staphyloma and pronounced choroidal atrophy were also noted. Although both conditions could occur as secondary changes in high myopia patients, such alterations usually require extensive periods to manifest. Given these findings, we propose that fundus changes, notably macular hypoplasia, may represent a hitherto feature of 48, XXYY syndrome [6], Table 1).

In 2010, Ottesen et al. examined the influence of sex chromosome genes and the gene dosage effect, potentially accounting for the increased stature [9]. A similar mechanism may apply to the genes linked to myopia, such as *MYP1*, *ARR3*, which are located on the X chromosome, suggesting a parallel between the genetic determinants of height and myopia [8, 10, 14].

In summary, we have documented the first reported macular dysplasia in a patient with 48, XXYY syndrome. Our report further expands the clinical spectrum of this disease. The patient’s decreased visual acuity could be attributed to the combined effects of high myopia and macular dysplasia. We postulate that these macular changes, particularly macular hypoplasia, could represent a previously unidentified aspect of 48, XXYY syndrome.

**Table 1:** Ocular clinical characteristics of 48, XXYY syndrome in previous studies

Patient	Ocular finding	Reference
12y,Male	Duane syndrome and mild myopia	Weis et al., 2011
8m,Male	Alternating exotropia, foveal hypoplasia and reduction of the foveal avascular zone	Chen et al., 2023
28y,Male	Lightly pigmented retinal pigment epithelium and choroid, peripapillary atrophy, pattern visual evoked potentials and pattern electroretinogram were undetectable bilaterally and high myopia	Karampelas et al., 2013

## Data Availability

All data generated or analysed during the current study are available from the corresponding author on reasonable request.
